# Efficacy of lobectomy versus segmentectomy for congenital lung malformations: a systematic review and meta-analysis

**DOI:** 10.1007/s00383-026-06362-1

**Published:** 2026-03-25

**Authors:** Shrouk F. Mohamed, Mohamed Abouegla, Mohamed Abouzeid, Amr Aljaradi, Aya Shahin, Ahmed Taha, Mohamed Eltaieb, Ayed Askar

**Affiliations:** 1 Faculty of Medicine, Alexandria, Egypt; 2 Mersey & West Lancashire Teaching Hospitals NHS Trust, Southport Hospital, Lancashire, England, UK; 3 Faculty of Medicine, Ain Shams University, Cairo, Egypt; 4 Faculty of Medicine, Al-Azhar University, Cairo, Egypt; 5 Faculty of Medicine, Assiut University, Asyut, Egypt; 6 Idlib University Hospital, Idlib, Syria

**Keywords:** Lobectomy, Segmentectomy, Congenital anomalies, Lung, Children

## Abstract

**Background:**

Congenital lung malformations (CLMs) are uncommon anomalies characterized by a broad clinical spectrum, from asymptomatic cases to severe respiratory distress. Surgical resection is often indicated; nevertheless, the optimal extent of resection is still debated, especially regarding lobectomy versus lung-sparing segmentectomy.

**Objective:**

This systematic review and meta-analysis aimed to compare the efficacy and safety of lobectomy versus segmentectomy in pediatric patients with CLMs.

**Methods:**

We searched databases, including PubMed, Scopus, Web of Science, and Cochrane, to identify papers comparing lobectomy with segmentectomy in pediatric patients with CLMs. The main outcome was the duration of hospital stay (LOS). Secondary outcomes included operative time, chest tube removal duration, pulmonary function indices, and postoperative complications. Random-effects meta-analyses were applied.

**Results:**

Nine retrospective observational studies involving infants and children were included. Meta-analysis showed no significant difference in LOS between lobectomy and segmentectomy (MD − 0.20, 95% CI − 0.83 to 0.44; *p* = 0.547). Operative time was significantly shorter with lobectomy (MD − 18.45, 95% CI − 27.21 to − 9.68; *p* < 0.0001). Chest tube removal time did not differ significantly between groups. Across all pulmonary function outcomes, no statistically significant differences were observed. Overall postoperative complications were similar between procedures (OR 0.96, 95% CI 0.58–1.61; *p* = 0.883).

**Conclusions:**

Lobectomy and segmentectomy demonstrate no differences in hospital duration, complications, or pulmonary function; nevertheless, lobectomy provides a shorter surgical time and diminished early air leakage without affecting overall postoperative results. High-quality, prospective studies are essential for clarifying long-term outcomes to improve surgical decision-making.

**Supplementary Information:**

The online version contains supplementary material available at 10.1007/s00383-026-06362-1.

## Introduction

Congenital lung malformations (CLMs) are rare developmental anomalies of the lung, including congenital pulmonary airway malformations (CPAM), bronchopulmonary sequestration, congenital lobar overinflation, bronchogenic cyst and isolated congenital bronchial atresia [1]. CLMs occur in 4 out of 10,000 live births [2]. Postnatal presentation ranges from an asymptomatic infant to respiratory failure. CLMs are typically diagnosed with antenatal ultrasonography and confirmed by chest CT angiography in the first few months of life [1, 2]. 

Accurate image-guided preoperative planning is required to identify suitable patients and to optimally prepare for segmentectomy [3]. Currently, preoperative planning in paediatric pulmonary surgery is most commonly aided by contrast-enhanced computed tomography imaging of the chest. Recent advances in imaging have enabled three-dimensional (3D) reconstruction and virtual reality (VR) visualization of patient-specific anatomy [4]. 

Recent surveys, reviews, and meta-analyses have sought to offer evidence-based guidance for managing patients with CLM. However, despite the growing body of literature, there remains no clear consensus on diagnostic, therapeutic, and follow-up strategies. The most controversial issue is the choice of approach, surgical resection versus observation, and the optimal timing of intervention for asymptomatic CPAM. Many surgeons favor early surgical management to prevent infections, lower the risk of degenerative changes, and allow for definitive histological evaluation of the lesion [5–8]. 

Surgical resection is considered the appropriate treatment option for symptomatic patients with CLMs, regardless of whether the diagnosis is made during fetal development or in childhood [9]. The objective of surgical intervention is to remove the affected lung tissue to alleviate symptoms, enhance pulmonary function in the remaining lung, prevent potential long-term complications, and facilitate compensatory lung growth [10]. However, the optimal type of surgical resection for pulmonary malformations continues to be a subject of discussion. There is a limited amount of data available that compares the outcomes of segmental lung-sparing resection with formal lobectomy for the treatment of congenital pulmonary malformations [11]. 

Therefore, the primary objective of this systematic review and meta-analysis is to evaluate the efficacy of Lobectomy versus Segmentectomy for congenital lung malformations.

## Materials and methods

Our systematic review and meta-analysis are reported according to the PRISMA (Preferred Reporting Items for Systematic Reviews and Meta-Analyses) 2020 [12]. The review protocol was registered on PROSPERO under registration number (CRD420251248237).

### Literature search and eligibility criteria

We performed a systematic search across the PubMed database using the keywords and MESH terms. We also searched each of SCOPUS, WEB OF SCIENCE, and COCHRANE databases using similar terms, as illustrated in Table S1 in the supplements.

Our inclusion criteria were randomized controlled trials and retrospective cohort studies reporting on patients with congenital lung malformations who underwent lobectomies versus segmentectomies. The eligibility criteria were structured according to a population–intervention–comparator–outcome (PICO) framework. The population comprised pediatric patients diagnosed with congenital lung malformations; the intervention was anatomical segmentectomy; the comparator was lobectomy; and the primary outcome was length of hospital stay (LOS), while secondary outcomes included operative time, time to chest tube removal, pulmonary function parameters, and overall postoperative complications.

We excluded case reports, narrative or systematic reviews, animal studies, and studies lacking a direct comparison of the two surgical approaches. No restrictions were placed on publication year, but only full-text articles in English were considered.

### Study selection and data collection

We used the Rayyan software for the study’s selection and screening. Two independent authors screened the papers separately, first by title and abstract, and then by full text. All disagreements were settled by group discussion.

### Data extraction

Two authors then extracted all pertinent data from the included studies after screening. Data extracted by the authors included Patients’ demographics (age, sex) and comorbidities; the duration and location of the study; and all outcomes listed above. Data was checked and confirmed by a third author.

### Quality assessment

The quality of the retrospective observational studies was checked using the Newcastle-Ottawa assessment tool by two separate authors, and all discrepancies and disputes were solved by group discussion.

### Statistical analysis

We used R software to perform the analysis for this study. Meta-analyses were performed using a random-effects model because of anticipated clinical and methodological heterogeneity across studies. For dichotomous outcomes, risk ratios with 95% confidence intervals (CIs) were calculated. Mean differences were used for continuous outcomes, and standardized mean differences were applied when studies reported different scales.

Statistical heterogeneity was evaluated with the I² statistic. Sensitivity analyses were conducted by excluding studies judged to be at high risk of bias. Publication bias was assessed using funnel plots and Egger’s test when studies were available for an outcome.

## Results

### Literature search

**Fig. 1:**
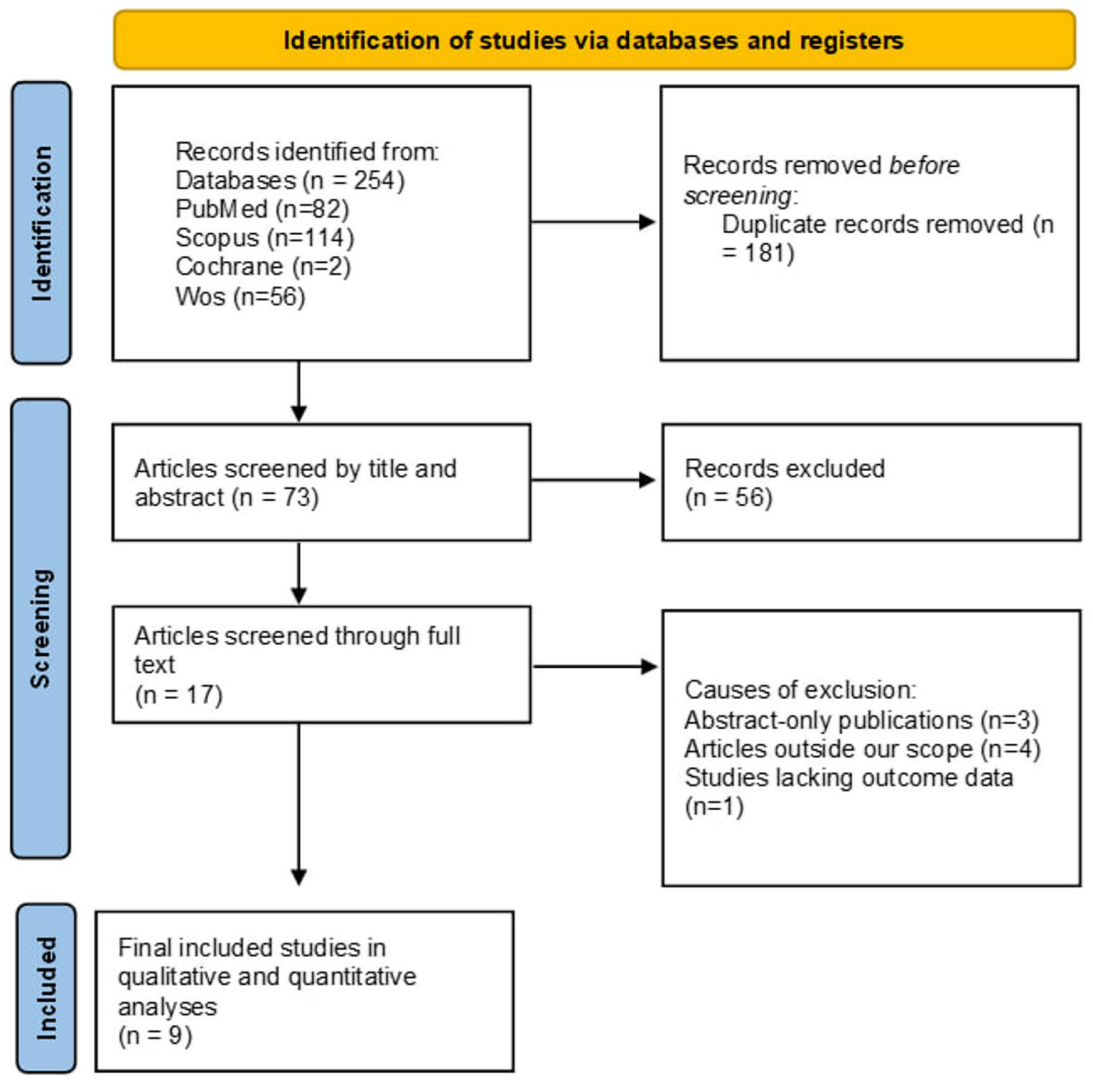
Prisma flow diagram

The systematic literature search yielded a total of records through the database search. Following the removal of duplicates, the remaining articles were evaluated based on their titles and abstracts. During the full-text screening step, we found that three studies were abstracts only, four studies were outside the review’s scope, and one study had incomplete data. Finally, nine studies that met eligibility criteria were included in the qualitative and quantitative synthesis [13–21], as illustrated in Fig. 1.

Study characteristics A total of nine retrospective observational studies comparing lobectomy and segmentectomy for CLMs were included in the quantitative synthesis. The population primarily consisted of infants and young children, with mean ages ranging from 0.58 months to 12.7 years at the time of surgery. Across studies, the lobectomy group consistently included more patients than the segmentectomy group. Reported male proportions ranged between 48 and 63% in studies that provided sex-specific data. Most of them were conducted in China. Several studies reported that segmentectomy provided comparable short-term and mid-term outcomes to lobectomy, particularly in terms of postoperative complications, length of hospital stay, and pulmonary function recovery. Two studies indicated that segmentectomy was associated with better preservation of short-term pulmonary function, while long-term pulmonary function remained normal in both groups. However, one large pediatric series reported a higher incidence of persistent air leakage following segmentectomy. Overall, the collective evidence suggests that segmentectomy is a feasible lung-sparing alternative to lobectomy in selected pediatric patients with CLMs, without compromising short-term safety, as summarized in Table 1.

**Table 1:** Characteristics of included studies

Study (year)	Country	Period of data collection	Groups	Sample size (n)	Age (mean ± SD)	Sex (male, n/%)	Key findings
Marinucci et al. 2023) 14	Italy	Between 2010 and 2020	Lobectomy	50	12.70 ± 11.90 years	30 (60%)	Lobectomy was associated with stable symptom remission and lower intra- and postoperative complications
			Segmentectomy	25	4.20 ± 11.60 years	12 (48%)	
Cheng et al. (2023) 18	China	January 2014 to January 2020	Lobectomy	361	6 ± 27 months	171	Thoracoscopic segmentectomy was safe and feasible with complication rates comparable to lobectomy
			Segmentectomy	206	6 ± 25.5 months	91	
Xi Huang et al. (2021) 15,19	China	January 2018 to March 2019	Lobectomy	19	4.46 ± 0.91 months	12 (63%)	Segmentectomy showed better short-term recovery of pulmonary function; long-term lung function was normal in both groups
			Segmentectomy	19	4.39 ± 0.78 months	12 (63%)	
Lee et al. (2016) 19	South korea	March 2011 and September 2015	Lobectomy	7	3.94 ± 2.31 months	16 (59.3%)	Segmentectomy preserved lung volume with low remnant lesion rates and similar outcomes to lobectomy
			Segmentectomy	20	NR	NR	
Polites et al. (2016) 21	USA	2008-2012	Lobectomy	634	1.6 ± 4 years	363	No difference in postoperative LOS or complications between lobectomy and sublobar resections
			Segmentectomy	486	1.8 ± 4.2 years	268	
Joo et al. (2023) 16	South korea	January 2010 and July 2020	Lobectomy	456	3.2 ± 2.5 years	NR	Postoperative outcomes were comparable; persistent air leakage was higher in segmentectomy
			Segmentectomy	85	NR	46 (55%)	
Bagrodia et al. (2014) 11	USA	March 2001 and September 2012	Lobectomy	26	0.58 ± 3.71 months	16 (62%)	Segmental resections were safely performed while conserving healthy lung tissue
			Segmentectomy	19	1.58 ± 1.5 months	10 (53%)	
Liao et al. (2024) 17	China	June 2021to June 2022	Lobectomy	9	8.67 ± 0.94 months	9	No significant difference in pulmonary function or complications between groups at 1 year
			Segmentectomy	21	10.76 ± 2.06 months	9	
He et al. (2025) 13	China	January 2021 and December 2023	Lobectomy	100	12 ± 9.3 months	NR	Segmentectomy was comparable to lobectomy in safety and efficacy when lesion boundaries were identifiable
			Segmentectomy	112	7.25 ± 5.3 months	NR	

*NR* not reported, *LOS* length of stay

### Quality assessment

The methodological quality of all included cohort studies was assessed using NOS. Overall, the studies demonstrated moderate to high methodological quality, with total NOS scores ranging from 7 to 9 stars. Most studies achieved the maximum score for selection, reflecting well-defined cohorts, reliable ascertainment of exposure through imaging and surgical records, and confirmation that outcomes were absent at baseline. Comparability scores were generally lower, as adjustment for confounders was limited in several studies, with only one multicenter propensity-matched study achieving full comparability. Outcome assessment was strong, with most studies employing objective outcome measurements, adequate follow-up durations, and low rates of loss to follow-up, as summarized in Table 2.

**Table 2:** Quality assessment of included cohort studies using the Newcastle–Ottawa scale (NOS)

Study (year)	Selection (4★)	Comparability (2★)	Outcome (3★)	Total Score (9★)	Overall quality
Cheng et al. (2022) 18	★★★★	★	★★★	**8/9**	High
Marinucci et al. (2023) 14	★★★★	★★	★★★	**9/9**	High
Lee et al. (2017) 19	★★★	★	★★★	**7/9**	Moderate
Polites et al. (2015) 21	★★★★	★★	★★	**8/9**	High
Joo et al. (2023) 16	★★★★	★	★★★	**8/9**	High
Liao et al. (2024) 17	★★★	★	★★★	**7/9**	Moderate
Bagrodia et al. (2014) 20	★★★	★	★★★	**7/9**	Moderate
He et al. (2025) 13	★★★★	★	★★	**7/9**	Moderate
Xi Huang et al. (2021) 15	★★★★	★★	★★	**8/9**	High

### Outcome assessment

#### Length of hospital Stay (LOS)

**Fig. 2:**
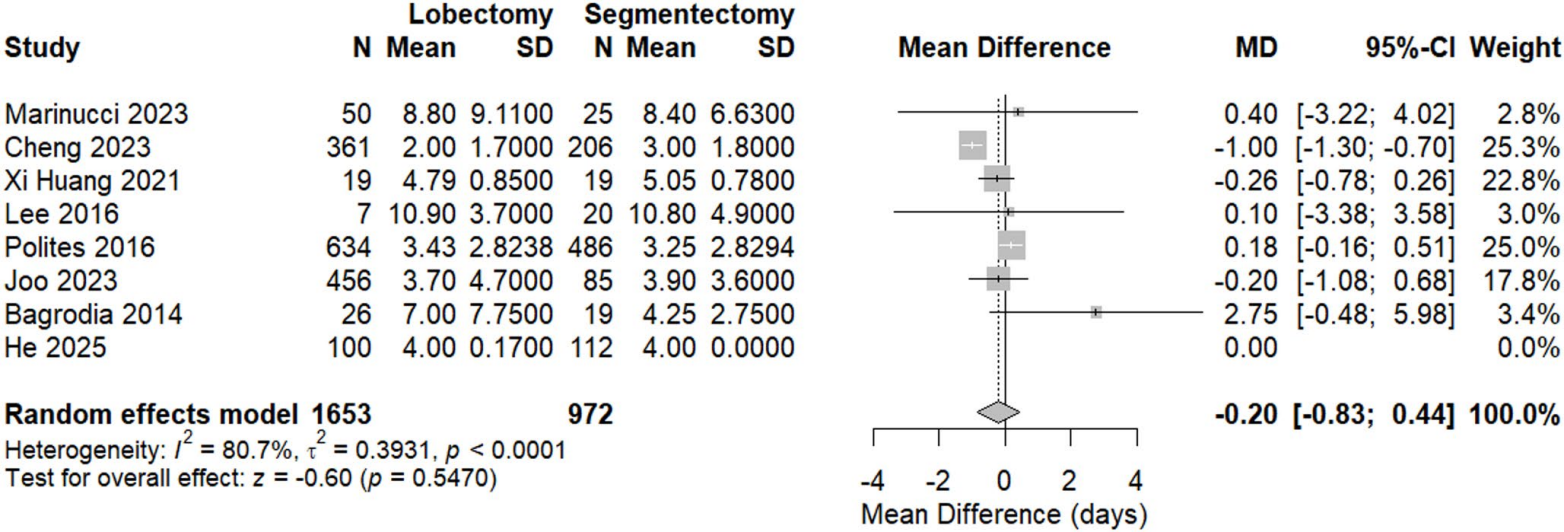
Forest plot of length of hospital stays

Meta-analysis was performed to assess the difference in LOS; the forest plot for LOS demonstrated no statistically significant difference between lobectomy and segmentectomy (MD = − 0.20, 95% CI [− 0.83 to 0.44], *p* = 0.547). Heterogeneity was high (I² = 80.7%), as shown in Fig. 2. Sensitivity analysis was performed by excluding the study by Cheng et al., which showed complete heterogeneity resolution, as shown in Figure S1 in the supplements.

Funnel plot asymmetry testing showed no evidence of publication bias (Egger’s t = 0.83, *p* = 0.444), indicating strong stability of the LOS result, as seen in Figure S2 in the supplements.

lobectomy.

#### Operative (Surgery) time

**Fig. 3:**
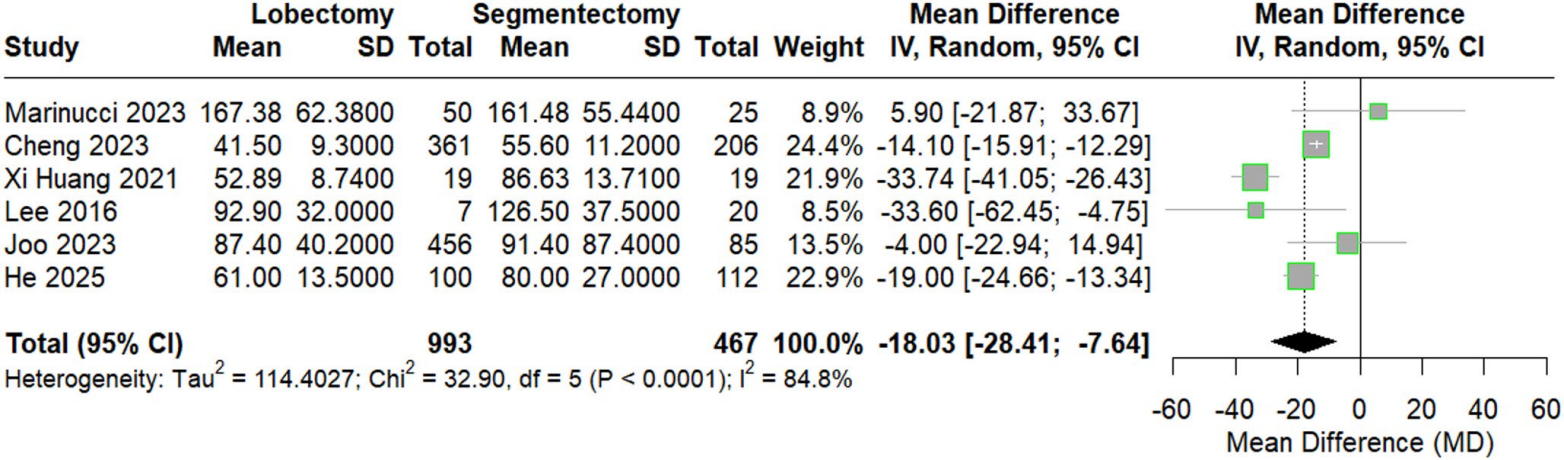
forest plot of surgery time

A pooled meta-analysis of operative time revealed a statistically significant difference between surgical techniques, showing that lobectomy performed in a shorter time (MD = − 18.45, 95% CI [− 28.41 to − 7.64], *p* < 0.0001). Between-study heterogeneity was substantial (I² = 84.8%), as shown in Fig. 3. Sensitivity analysis was performed by excluding the study by Xi Huang et al., which helped reduce heterogeneity, with I² = 47%, as shown in Figure S3 in the supplementary materials.

Publication bias assessment showed no significant bias (Egger’s t = − 65, *p* = 0.5526), as shown in Figure S4 in the supplements.

#### Chest tube removal

**Fig. 4:**
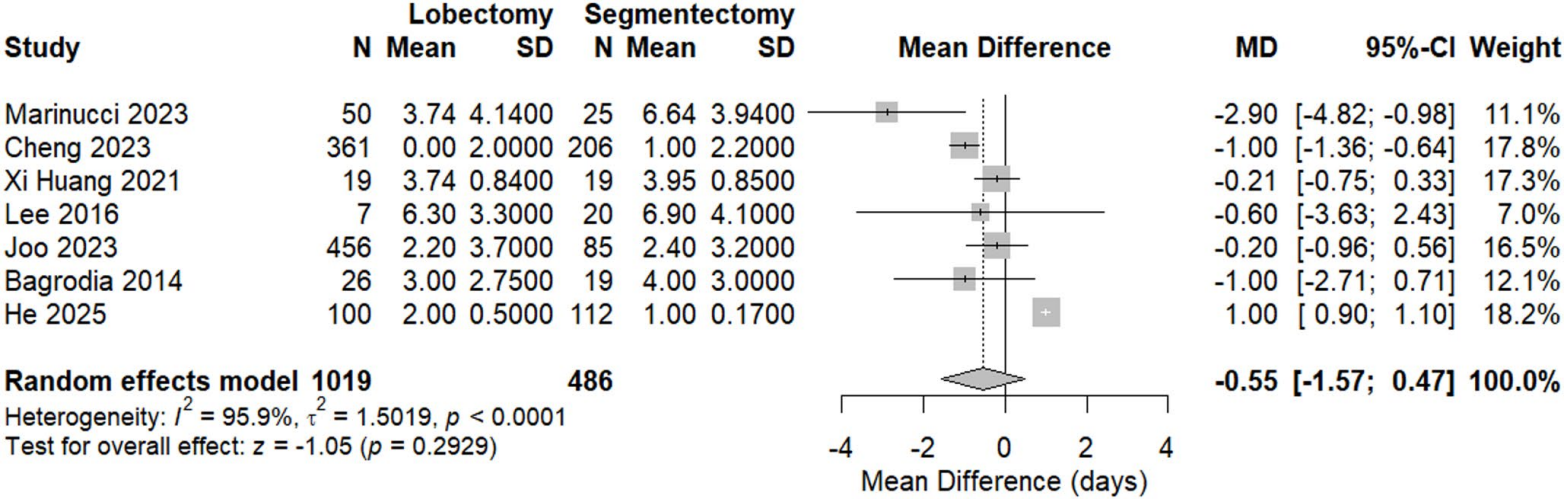
forest plot of chest tube removal

We found no statistically significant difference between the two groups regarding the time taken to remove chest tubes after surgery (MD = − 0.55, 95% CI [− 1.57 to 0.47], *p* = 0.2929). Between-study heterogeneity was high (I² = 95.9%), as shown in Fig. 4. Sensitivity analysis was performed by excluding the study by He et al., which helped reduce heterogeneity, with I² = 60.2%. Additionally, a change in the context of results following sensitivity analysis supports the association between lobectomy and lower time, as shown in Figure S5 in the supplementary materials.

Publication bias assessment approached but did not reach significance (Egger’s t = − 2.38, *p* = 0.063), suggesting a possible small-study effect for this outcome (Figure S6 in the supplements).

#### Pulmonary function outcomes

Across all pulmonary function indices, the forest plots demonstrated no statistically significant differences between lobectomy and segmentectomy. The confidence intervals overlapped neutral effect thresholds in all comparisons, indicating comparable postoperative lung function preservation between the two surgical approaches.

#### Tidal volume (VT)

No significant difference was observed between lobectomy and segmentectomy (MD = − 0.06, 95% CI [− 0.27 to 0.14], *p* = 0.5337). There was low heterogeneity with I² = 11%, as seen in Figure S7 in the supplements.

#### Inspiratory time to expiratory time ratio (TI/TE)

There was no statistically significant difference between the two surgical techniques (MD = 0.01, 95% CI [− 0.06 to 0.08], *p* = 0.8152). There was no heterogeneity, as indicated by an I² value of 11%, as shown in Figure S8 in the supplements.

#### Time to peak tidal expiratory flow to total expiratory time ratio (TPTEF/TE)

Pooled results showed no significant difference (MD = 2.34, 95% CI [− 2.44 to 7.12], *p* = 0.3375). There was high heterogeneity, as indicated by an I² value of 95.1%, as shown in Figure S9 in the supplements.

#### Volume of peak expiratory flow to expired volume ratio (VPEF/VE)

Similarly, no significant difference was identified (MD = 1.58, 95% CI [− 1.73 to 4.88], *p* = 0.3497). There was high heterogeneity, as indicated by an I² value of 93.3%, as shown in Figure S10 in the supplements.

#### Overall postoperative complications

**Fig. 5:**
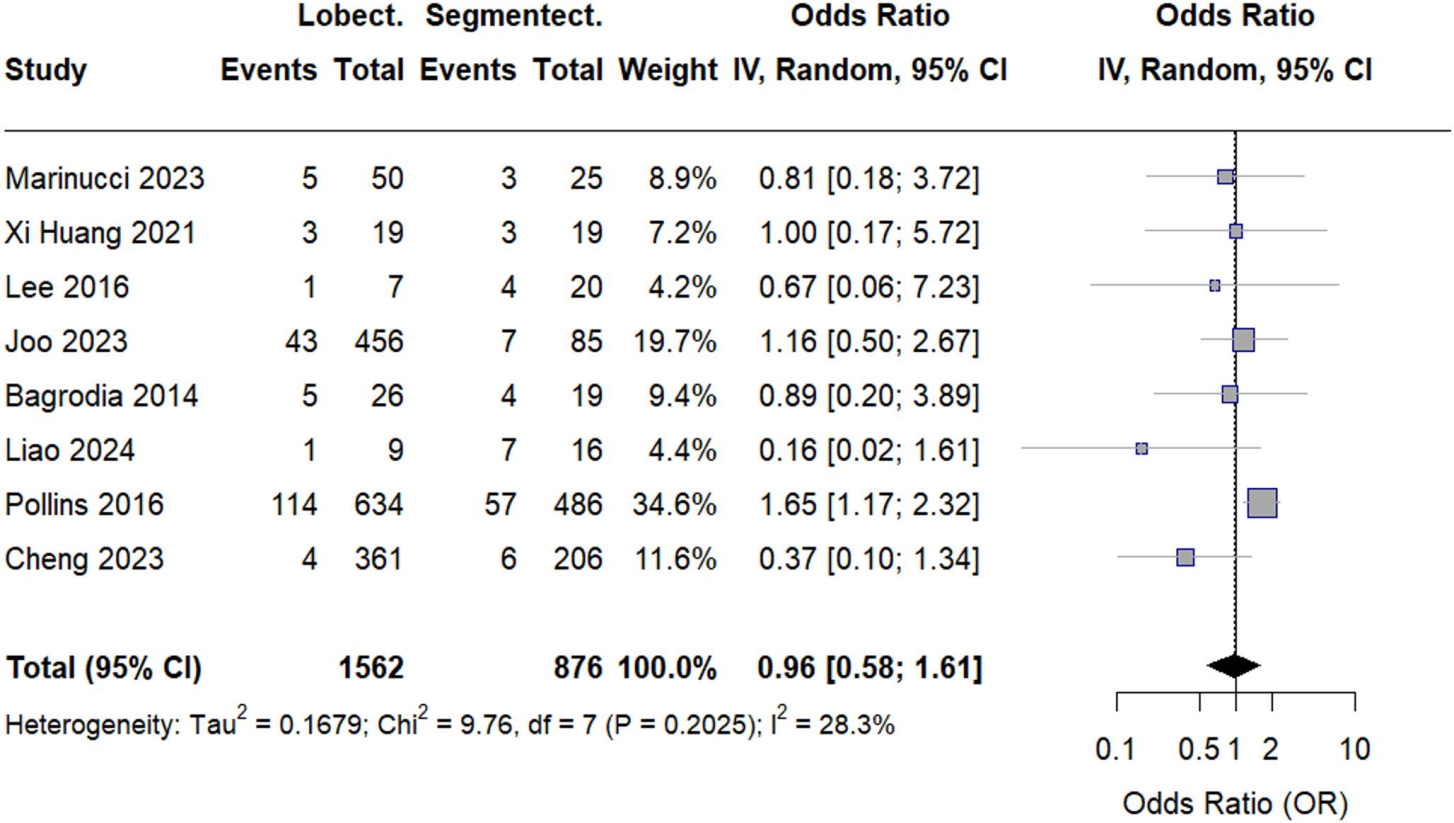
Forest plot of complication rate

The pooled analysis of overall complications showed no statistically significant difference between groups, as indicated in the forest plot (OR = 0.96, 95% CI [0.58–1.61], *p* = 0.8827). Heterogeneity for complications was low (I² = 28.3%), as shown in Fig. 5. Sensitivity analysis was performed by excluding the study by Cheng et al., which helped resolve heterogeneity, with an I² value of 0%, as shown in Figure S11 in the supplements.

However, publication bias was detected for this outcome (Egger’s t = − 3.89, *p* = 0.008), indicating that the pooled complication effect may be influenced by small-study effects or selective reporting, as seen in Figure S12 in the supplements.

#### Pneumonia

Two studies were included in this analysis; the forest plot analysis showed no statistically significant difference in postoperative pneumonia between lobectomy and segmentectomy (OR = 0.47, 95% CI [0.07–3.27], *p* = 0.4427). There was no observed heterogeneity, as seen in Figure S13 in the supplements.

#### Bleeding

Three studies examined the incidence of bleeding, showing no significant intergroup difference (OR = 2.96, 95% CI [0.38–22.92], *p* = 0.2989). Low heterogeneity was observed (I² = 25.9%), as shown in Figure S14 in the supplementary materials. Sensitivity analysis was performed by excluding the study by Polites et al., which resolved heterogeneity, as shown in Figure S15 in the supplementary materials.

#### Pneumothorax

Three studies examined the incidence of pneumothorax, showing no significant intergroup difference (OR = 1.22, 95% CI [0.36–4.12], *p* = 0.7483). No heterogeneity was observed (I² = 0%), as shown in Figure S16 in the supplementary materials.

#### Infection

Postoperative infection rates showed a statistically significant difference between lobectomy and segmentectomy across all included studies (OR = 1.06, 95% CI [0.10–10.68], *p* = 0.924). No heterogeneity was observed (I² = 0%), as shown in Figure S17 in the supplementary materials.

#### Pleural effusion

No statistically significant difference was observed in pleural effusion rates (OR = 0.31, 95% CI [0.03–2.99], *p* = 0.3125). No heterogeneity was observed (I² = 0%), as shown in Figure S18 in the supplementary materials.

#### Air leakage

Three studies examined the incidence of air leakage, showing a significant difference between the two groups, favoring lobectomy (OR = 0.30, 95% CI [0.11–0.83], *p* = 0.0201). Low heterogeneity was observed (I² = 21.6%), as shown in Figure S19 in the supplementary materials. Sensitivity analysis was performed by excluding the study by Joo et al., which resolved heterogeneity and altered the context of the results, showing no difference between the groups, as shown in Figure S20 in the supplementary materials.

## Discussion

CLMs are rare developmental anomalies that affect the life of neonates. Early surgical intervention is often needed for such cases; however, there is no standardized protocol for the extension of surgical resection between lobectomy and segmental resection [22, 23]. This study examined the advantages and safety of segmentectomy versus lobectomy as a feasible lung-sparing procedure in paediatric patients with CLMs. Advances in imaging and minimally invasive techniques have led to an increased interest in segmentectomy; however, lobectomy remains the gold standard in children with CLMs.

Numerous studies reported that lobectomy is a good option to prevent malignant transformation in congenital anomalies [24, 25]. Our results showed no superiority of segmentectomy over lobectomy, where most of the results showed no significant differences.

Regarding hospital stay, there was no difference between the two techniques. This is consistent with findings from previous studies, supporting that postoperative recovery trajectories are equivalent between the two techniques, regardless of resection extent [16, 26]. 

Our results showed that lobectomy was performed in a shorter time as compared to segmentectomy. Consistent with us, Joo et al. demonstrated that segmentectomy is associated with a longer operation time in comparison to lobectomy [16]. Also, Huang et al. showed a similar outcome to our study. While the increased duration of segmentectomy is due to the technical complexity in delineating segmental planes and preserving intersegmental structures, which makes it acceptable from a safety standpoint, as no corresponding increase in perioperative complications or hospital stay was observed [15].

Our findings showed no difference in possible complications like postoperative infection, pneumothorax, bleeding and pleural effusion. Similarly, Polites et al. found no differences in complications between the two groups [21]. In contrast to our findings, Marinucci et al. showed that lobectomy has been associated with fewer both intra-operative and post-operative complications than segmentectomy [14]. 

Importantly, air leakage was more common following segmentectomy, although this association disappeared upon sensitivity testing. This transient significance is consistent with the technique-dependent nature of air leaks, as described by Joo et al., which highlights the importance of meticulous intersegmental plane management and fissure sealing during pediatric resections [16]. Johnson et al. and Fascetti-Leon et al. also reported that the incidence of complications such as bleeding and air leakage after segmentectomy was high, with high reoperation rates [27, 28]. 

Maintaining pulmonary function after surgery is a major concern for children because their lungs may develop back to normal. Our study found no significant difference between lobectomy and segmentectomy in terms of pulmonary function indices such as VT, TI/TE ratio, TPTEF/TE, and VPEF/VE [29]. Liu et al. and He et al. both found that the lung functions remain normal in most patients following lobectomy [13, 30]. 

Additionally, McBride et al. indicated that children who underwent lobectomy in infancy attained nearly normal total lung volumes, despite the excision of around 8–45% of lung tissue [31]. Frenckner and Freyschuss similarly showed that patients who had lobectomies and were followed for up to 11 years had total lung volumes that were close to 90% of what would be expected for normal lungs. This suggests that the lungs grew to make up for the loss of a lobe [32]. 

### Study limitations

This meta-analysis has several limitations that should be stated. First, the included articles were retrospective observational studies, as we could not find any relevant clinical trials. There were also variations in the experience of the surgeons, the surgical techniques used (thoracoscopic vs. open), and the protocols for postoperative care, which could have affected the results of the studies that were included. The individuals in the study were all different from each other, and all had different congenital lung problems, which may make it hard to apply the results to other people. Multiple analyses demonstrated moderate to high statistical heterogeneity. Additional limitations include very small sample sizes in some studies, uneven documentation of long-term pulmonary function results, and variation in follow-up time. There is a need for prospective, multicenter randomized studies to establish definitive equivalence between segmentectomy and lobectomy for CLMs.

## Conclusion

Although there were no significant differences between lobectomy and segmentectomy in length of hospital stay, chest tube removal time, pulmonary function outcomes, or overall postoperative complications, lobectomy remains a better option for congenital lung anomalies as it takes a shorter operation time. Further studies are needed to support lesion-tailored resection.

## Supplementary Information

Below is the link to the electronic supplementary material.

Supplementary Material 1

## Data Availability

All data generated or analyzed during this study are included in this published article.
