# Supplementary material for: Efficacy of lobectomy versus segmentectomy for congenital lung malformations: a systematic review and meta-analysis
Source: Pediatr Surg Int. 2026 Mar 25;42(1):165. doi: 10.1007/s00383-026-06362-1 (PMC13018017; doi:10.1007/s00383-026-06362-1)
Supplement: Supplementary file 1 — Supplementary Material 1 [file 383_2026_6362_MOESM1_ESM.docx]

Table S1. Search strategy for each database

| Database | Search strategy | Number of results |
| --- | --- | --- |
| PubMed | (("Congenital Lung Malformations"[Mesh] OR "congenital lung malformation*" OR "congenital pulmonary airway malformation*" OR CPAM OR CCAM OR "pulmonary sequestration" OR "bronchogenic cyst" OR "congenital lobar emphysema") AND (segmentectomy OR "segmental resection" OR "sub-lobar resection" OR "lung-sparing surgery") AND (lobectomy OR "thoracoscopic surgery" OR VATS OR "video-assisted thoracic surgery") AND (child* OR pediatric* OR infant* OR neonate*)) | 82 |
| Scopus | (("Congenital Lung Malformations" OR "congenital lung malformation*" OR "congenital pulmonary airway malformation*" OR CPAM OR CCAM OR "pulmonary sequestration" OR "bronchogenic cyst" OR "congenital lobar emphysema") AND (segmentectomy OR "segmental resection" OR "sub-lobar resection" OR "lung-sparing surgery") AND (lobectomy OR "thoracoscopic surgery" OR VATS OR "video-assisted thoracic surgery") AND (child* OR pediatric* OR infant* OR neonate*)) | 114 |
| Web of Science | (("Congenital Lung Malformations" OR "congenital lung malformation*" OR "congenital pulmonary airway malformation*" OR CPAM OR CCAM OR "pulmonary sequestration" OR "bronchogenic cyst" OR "congenital lobar emphysema") AND (segmentectomy OR "segmental resection" OR "sub-lobar resection" OR "lung-sparing surgery") AND (lobectomy OR "thoracoscopic surgery" OR VATS OR "video-assisted thoracic surgery") AND (child* OR pediatric* OR infant* OR neonate*)) | 56 |
| Cochrane | (("Congenital Lung Malformations" OR "congenital lung malformation" OR "congenital pulmonary airway malformatio*" OR CPAM OR CCAM OR "pulmonary sequestration" OR "bronchogenic cyst" OR "congenital lobar emphysema") AND (segmentectomy OR "segmental resection" OR "sub-lobar resection" OR "lung-sparing surgery") AND (lobectomy OR "thoracoscopic surgery" OR VATS OR "video-assisted thoracic surgery") AND (child OR pediatric OR infant* OR neonate)) | 2 |


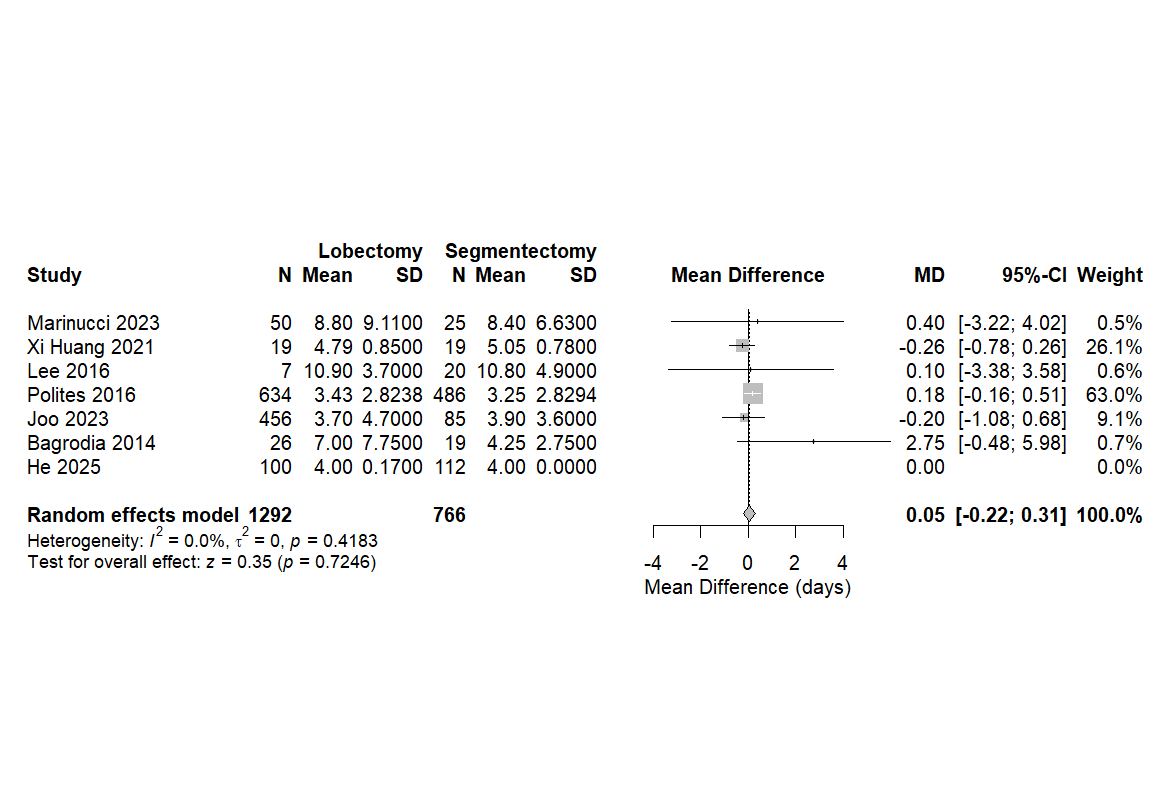


Figure S1: sensitivity analysis of length of hospital stays.


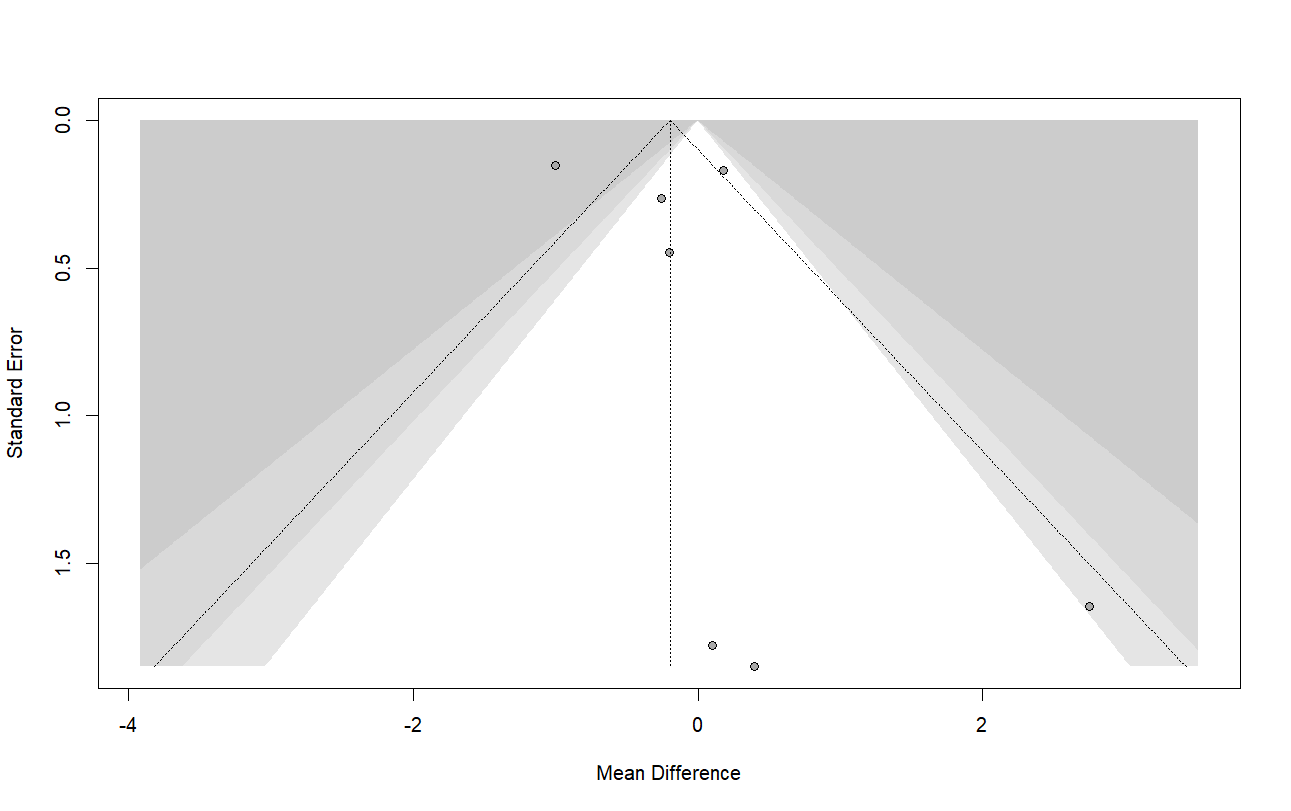


Figure S2: Funnel plot of of length of hospital stays.


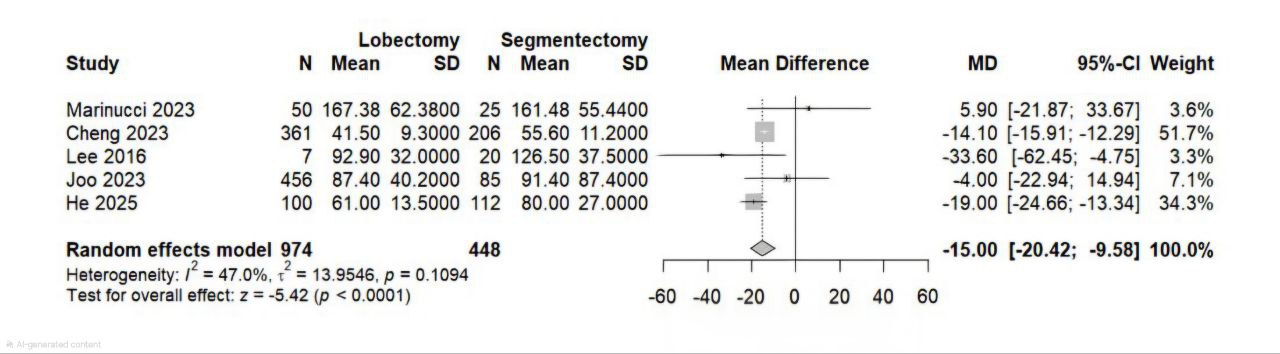


Figure S3: sensitivity analysis of surgery time.


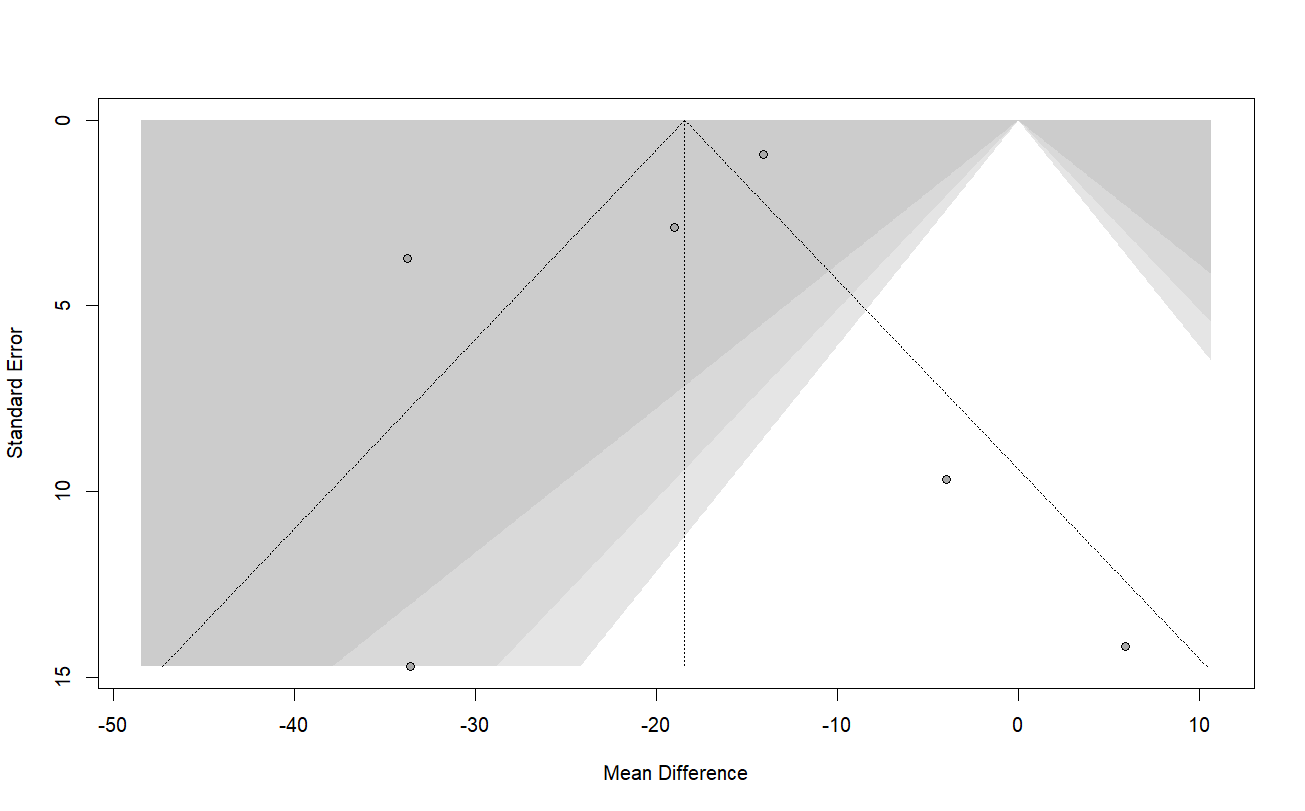


Figure S4: funnel plot of surgery time


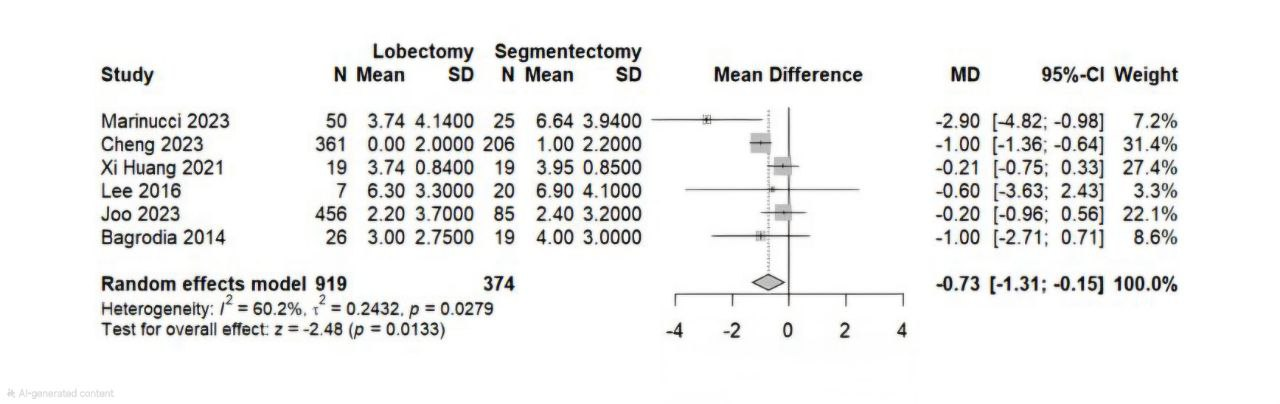


Figure S5: sensitivity analysis of chest tube removal.


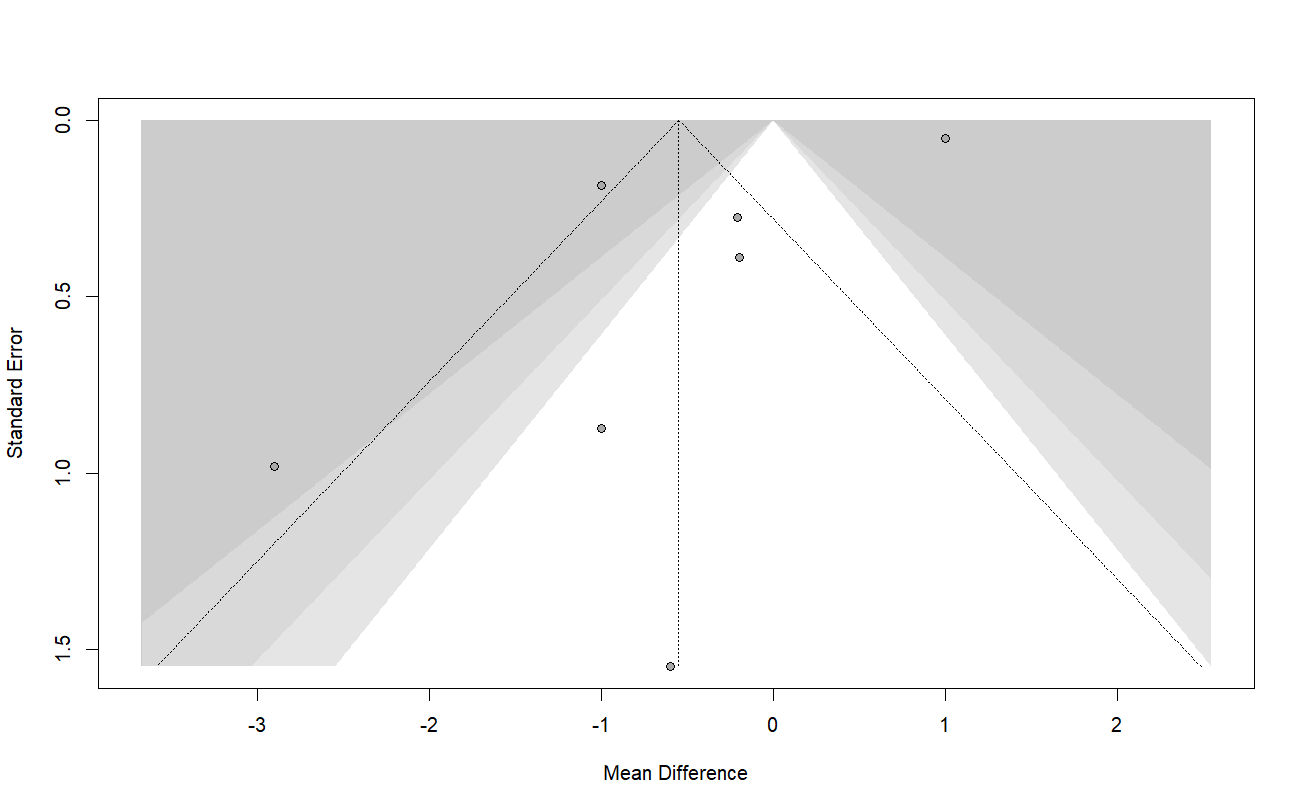


Figure S6: funnel plot of chest tube removal.


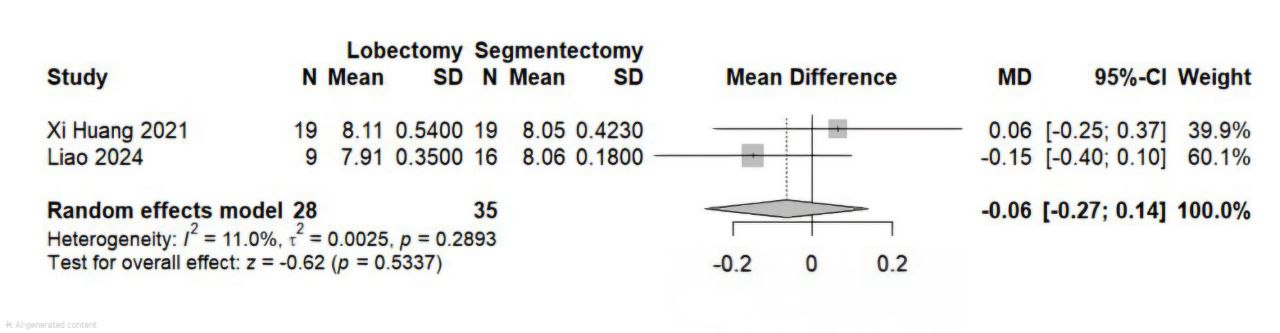


Figure S7: forest plot of VT.


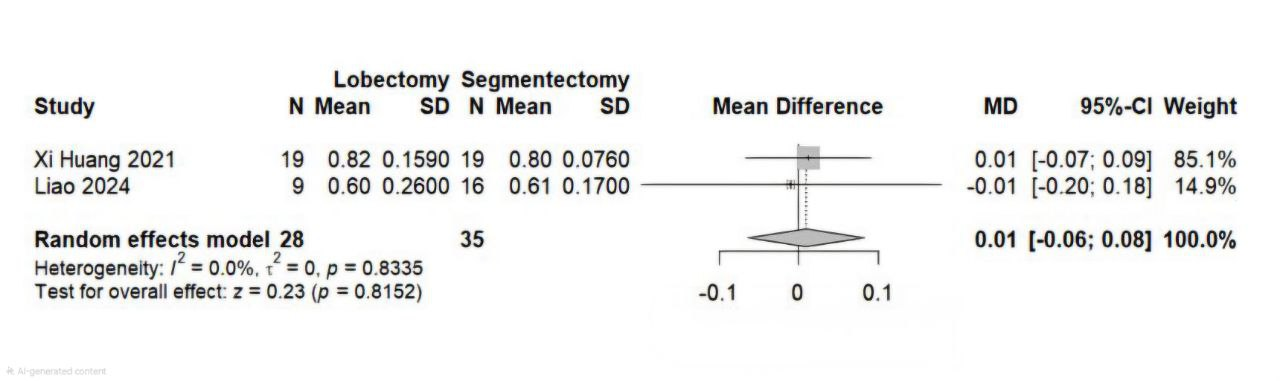


Figure S8: forest plot of TI TE.


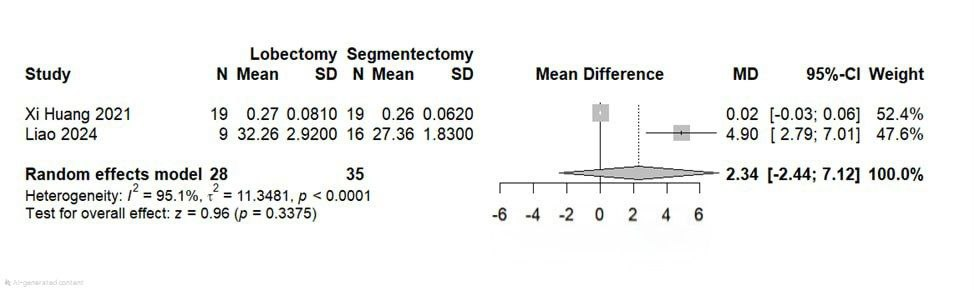


Figure S9: forest plot of TPTEF TE


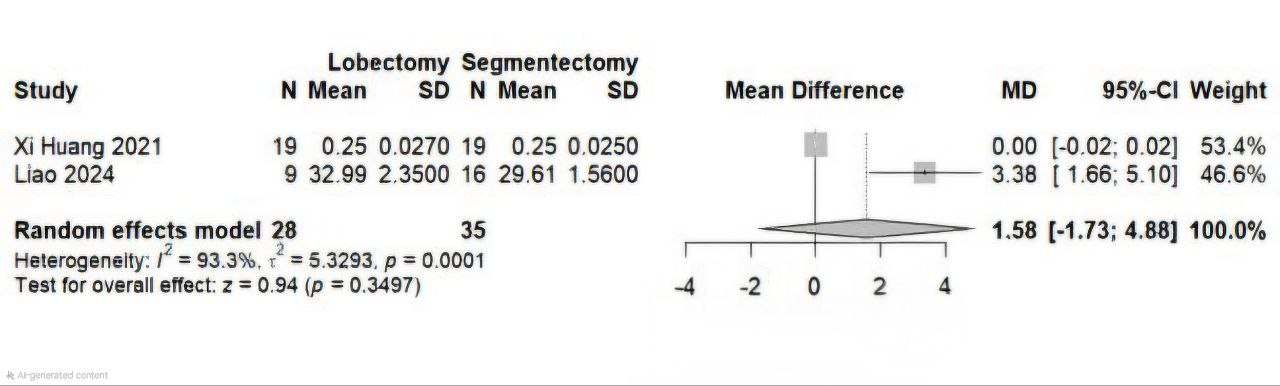


Figure S10: Forest plot of VPEF VE.


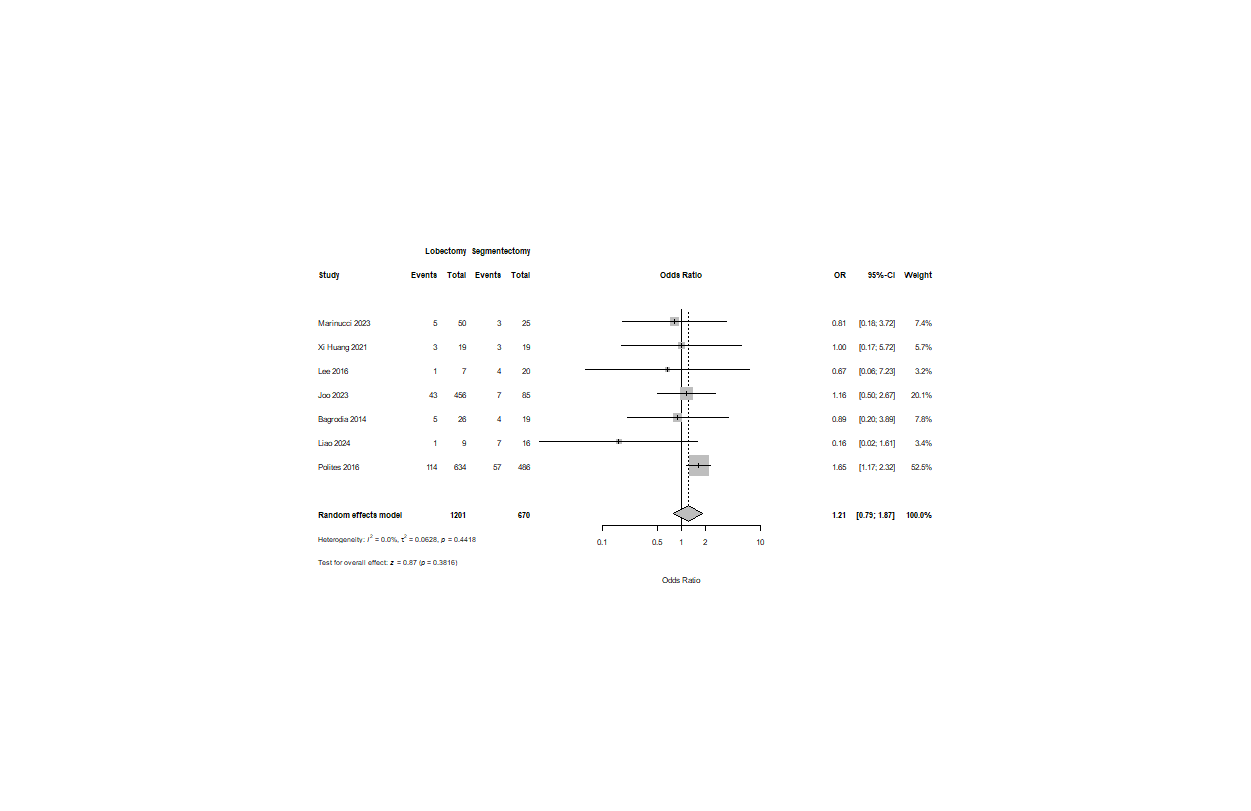


Figure S11: sensitivity analysis of the complication rate.


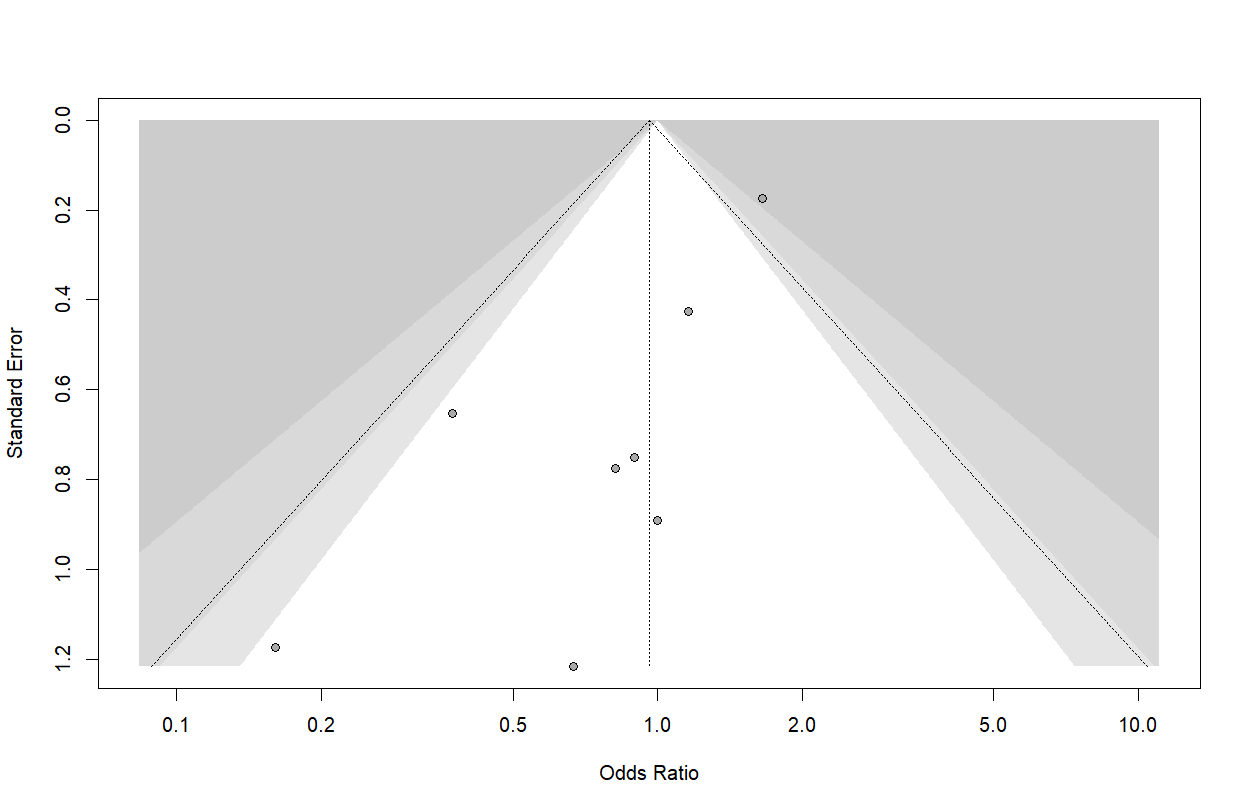


Figure S12: Funnel plot of complication rate.


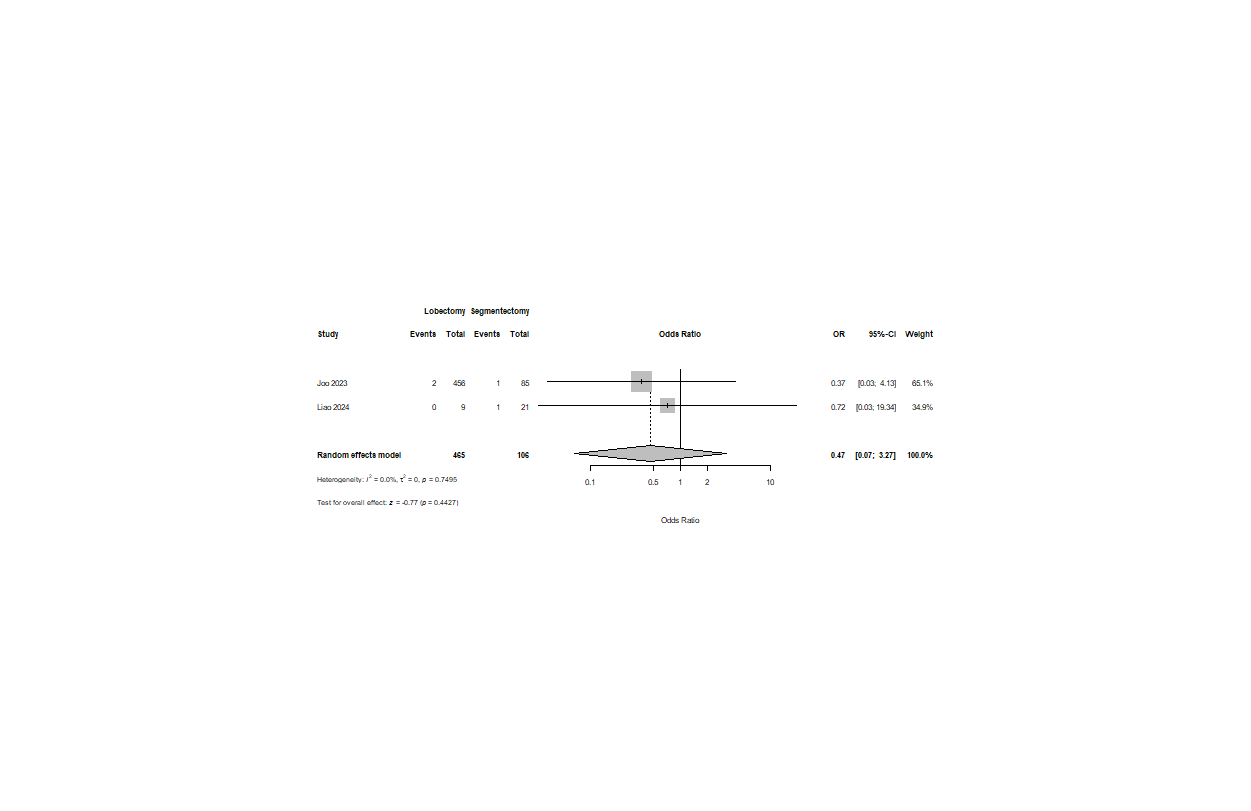


Figure S13: forest plot of pneumonia.


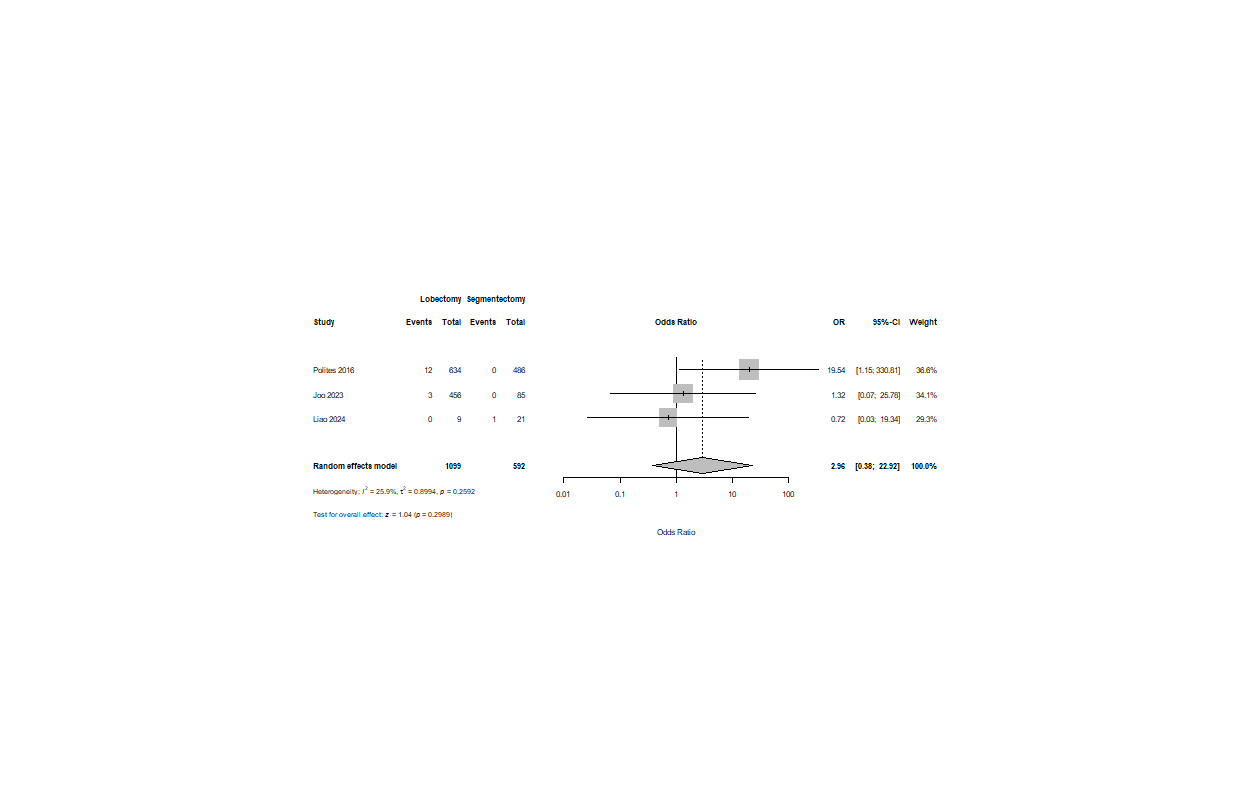


Figure S14: forest plot of bleeding.


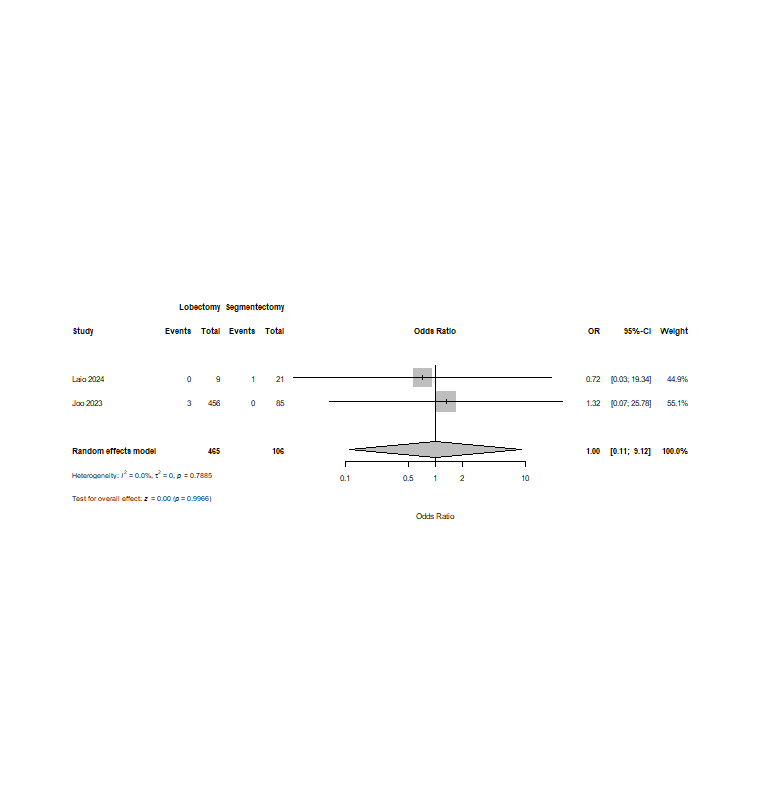


Figure S15: sensitivity analysis of bleeding.


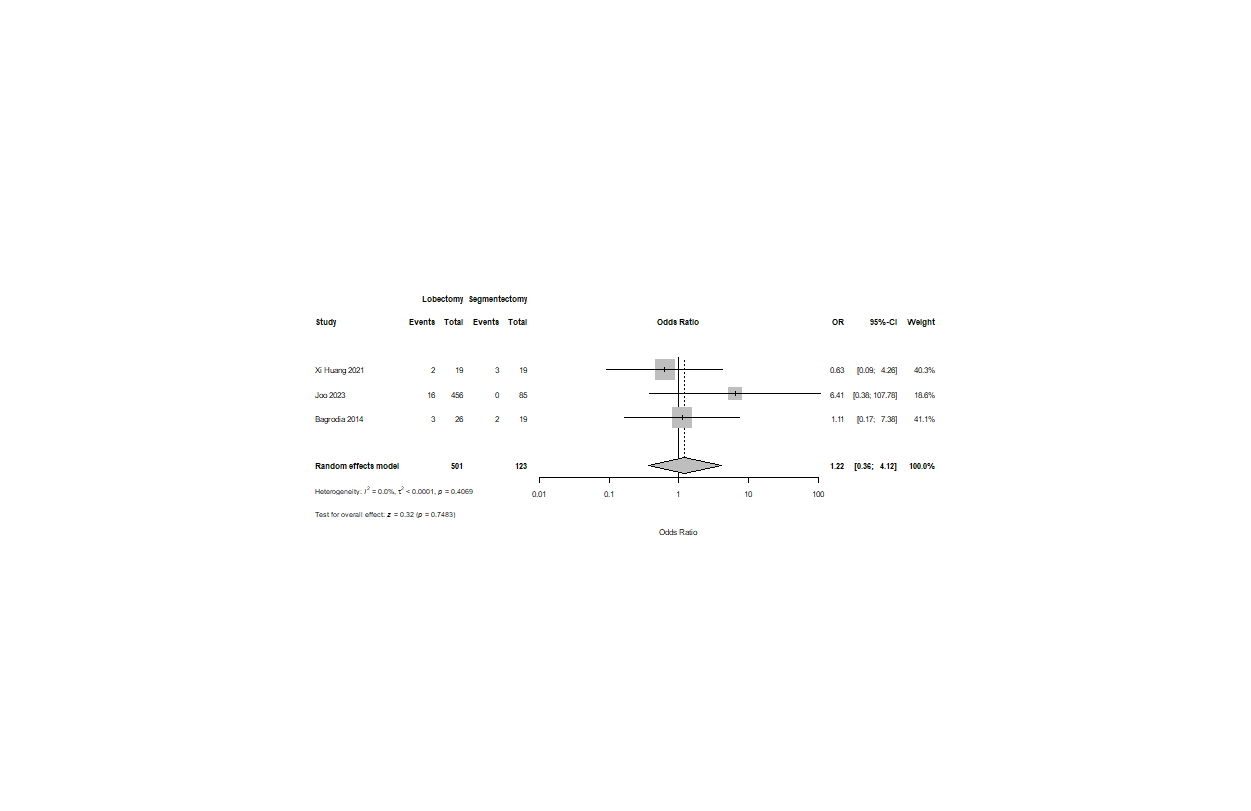


Figure S16: forest plot of pneumothorax.


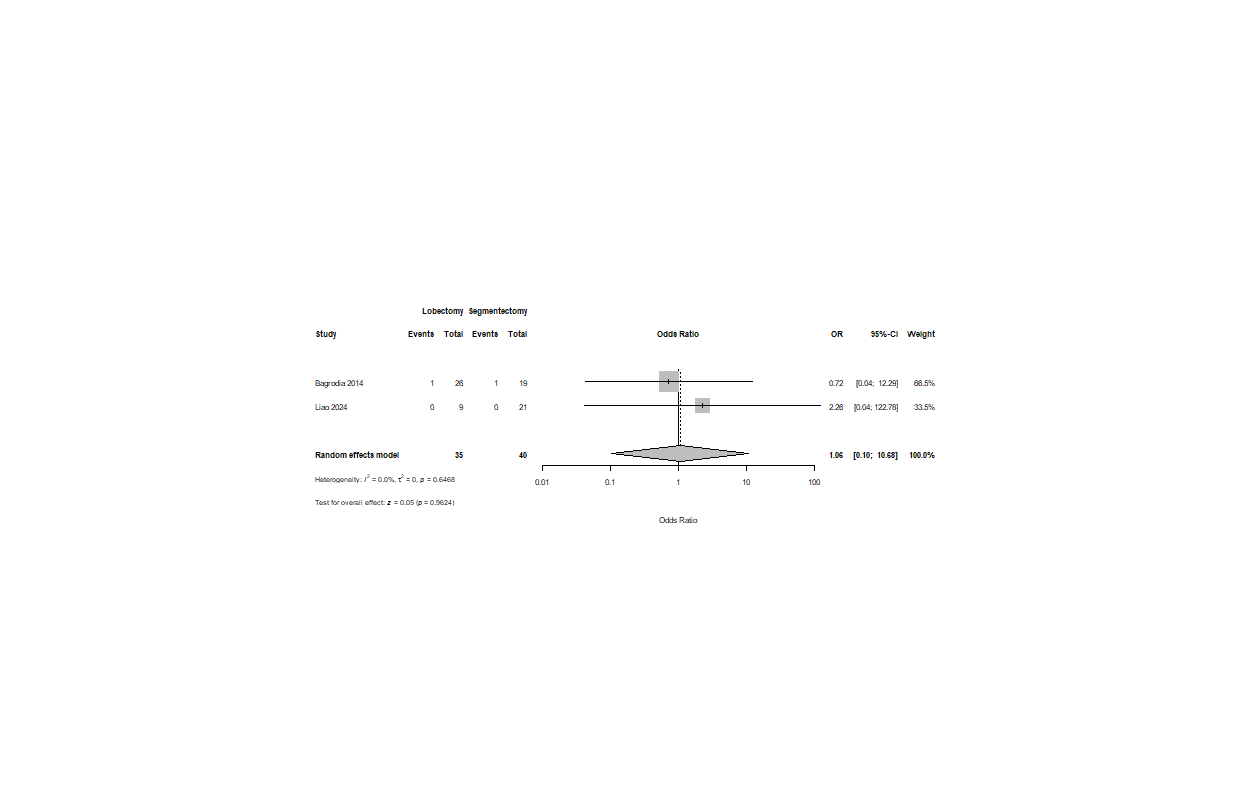


Figure S17: forest plot of infection.


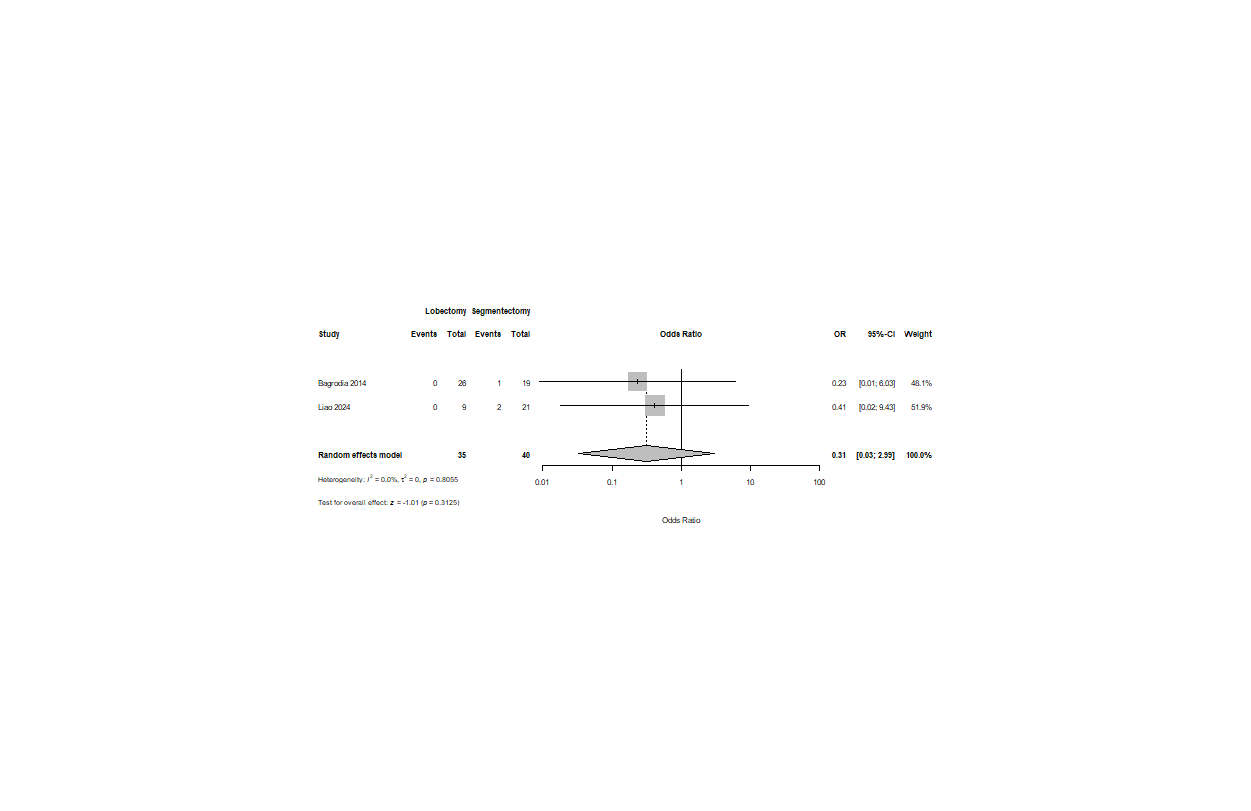


Figure S18: forest plot of pleural effusion.


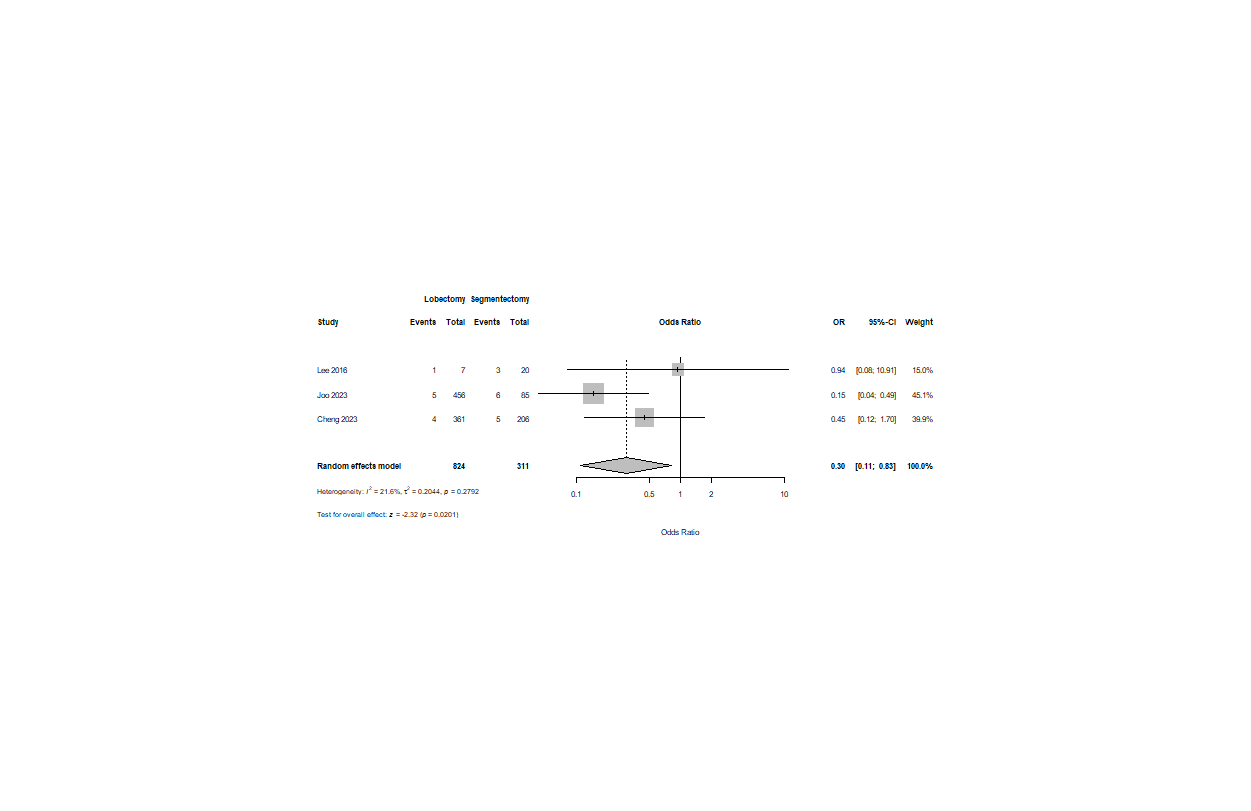


Figure S19: forest plot of air leakage.


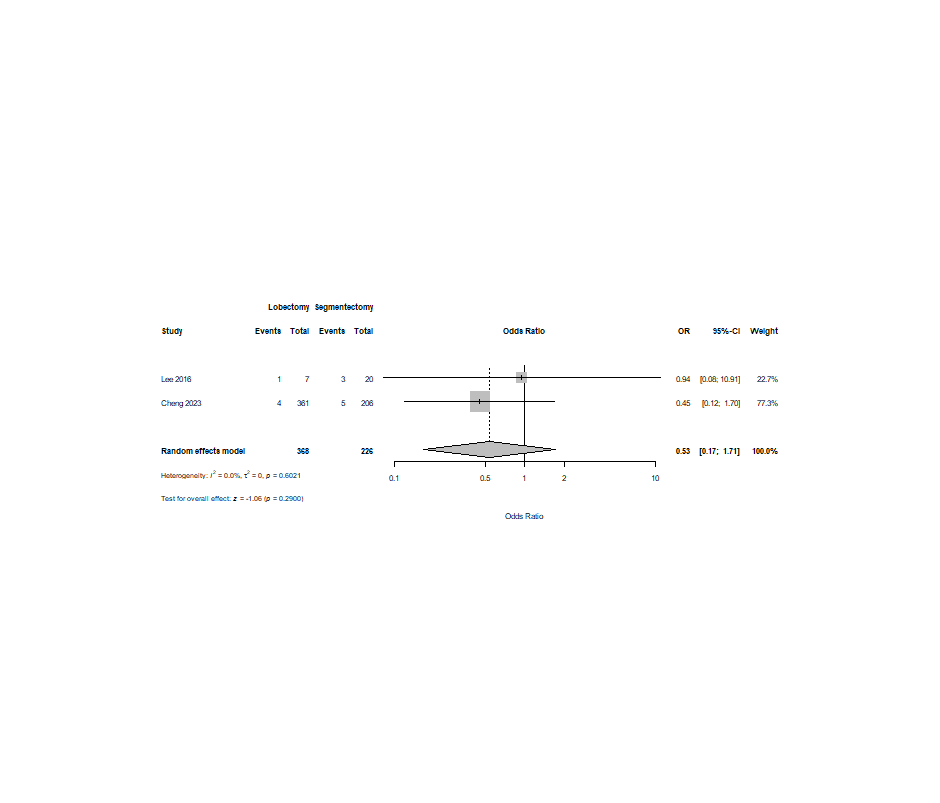


Figure S20: sensitivity analysis of air leakage.
